# Choosing the “Ideal” Oral Dosage Form for Pediatric Patients: Parents’ Perspectives on Children’s Preferences with a Focus on Orodispersible Tablets

**DOI:** 10.3390/children12091187

**Published:** 2025-09-05

**Authors:** Yana Gvozdeva, Radiana Staynova

**Affiliations:** 1 Department of Pharmaceutical Technology and Biopharmacy, Faculty of Pharmacy, Medical University of Plovdiv, 4002 Plovdiv, Bulgaria; yana.gvozdeva@mu-plovdiv.bg; 2 Research Institute, Medical University of Plovdiv, 4002 Plovdiv, Bulgaria; 3 Department of Organisation and Economics of Pharmacy, Faculty of Pharmacy, Medical University of Plovdiv, 4002 Plovdiv, Bulgaria

**Keywords:** pediatrics, age-appropriate, orodispersible tablets, excipients, taste, parents

## Abstract

*Background*: Developing suitable dosage forms presents multiple challenges, such as ensuring the medication can be easily swallowed by young children, mixed with a small amount of food or liquid, and effectively taste-masked. There is no standardized guidance on pediatric dosage forms, taste preferences, or acceptable excipients, often resulting in costly delays due to required toxicology studies. Additionally, regulatory considerations around bioequivalence may necessitate further discussions between industry and regulatory authorities. *Objective*: This research aimed to investigate and analyze Bulgarian parents’ perspectives on their children’s preferences regarding different oral dosage forms, with a particular emphasis on orodispersible tablets (ODTs). Additionally, challenges related to the development of age-appropriate formulations were comprehensively discussed. *Methods*: A cross-sectional, online questionnaire-based study was conducted among 303 parents in Plovdiv, the second-largest city in Bulgaria, between January and March 2021. *Results*: The majority of parents (78.2%) reported no difficulties in administering medication to their child. Liquids were identified as the most preferred oral dosage form (68.3%), followed by tablets (21.8%). With respect to the importance of taste, most parents indicated that it is a very important factor influencing their child’s acceptance of medication. Although 249 parents stated that they were familiar with ODTs, only 11.2% reported that their child had previously taken ODTs. *Conclusions*: The results of our study show that the taste of the dosage form is a leading factor in child acceptability. The sweet fruit flavor was a favorite among children. Parental attitudes toward ODTs were strongly positive, with 91.1% indicating a preference for their child to receive ODTs rather than conventional tablets.

## 1. Introduction

Pediatric patients are typically defined as individuals from birth up to either 16 or 18 years of age, depending on regional regulations [1]. They differ from adults in many aspects of pharmacotherapy, including the ability to administer drugs related to drug toxicity and taste preferences. The implementation of Paediatric Regulation (EC) No. 1901/2006 has significantly increased the emphasis on developing medicines specifically for children, highlighting the need for age-appropriate formulations [2]. This remains challenging, as numerous unresolved questions still confront pharmaceutical companies, academic researchers, and regulatory authorities [3]. Pediatric drug formulations must correspond to the age, weight and physiological condition of the child [4]. To address this, the World Health Organization created a pediatric essential medicines list that highlights priority formulations designed for children [5]. The European pediatric regulation has played a pivotal role in achieving progress, mandating that pediatric strategies be integrated into all drug development programs. However, developing pediatric medicines is a complex, resource-intensive, and time-consuming process [3].

Oral administration is the most commonly recommended route as it offers a convenient and non-invasive way to deliver medication to pediatric patients across various age groups. With a wide range of oral dosage forms available, this route is generally preferred [6].

Pediatric patients differ from adults not only in physiology but also in potential routes of drug absorption. The lack of age-appropriate oral dosage forms for children has been associated with significant clinical consequences [7]. Notable examples include pediatric fatalities resulting from choking on albendazole tablets and adverse outcomes due to the use of excipients such as benzyl alcohol or diethylene glycol in sulfonamide formulations [4]. Numerous studies have shown the growing importance of the type of dosage form and the development of patient-oriented dosage forms, which meet the specific requirements of certain target groups of patients [8].

Pediatric formulations should enable accurate dosing across a wide range of ages and body weights [9]. The European Medicines Agency (EMA) has established specific guidelines for the development and authorization of pediatric dosage forms [10]. These guidelines recommend that manufacturers provide both oral liquid and solid dosage forms of a given drug or ensure that formulations can be modified to meet individual patient needs. As already mentioned, the oral route remains the most preferred method of drug administration in pediatric populations [11]. Notably, studies have reported substantial advancements in the development of oral pediatric formulations for the treatment of cardiovascular diseases [12].

**Figure 1:**
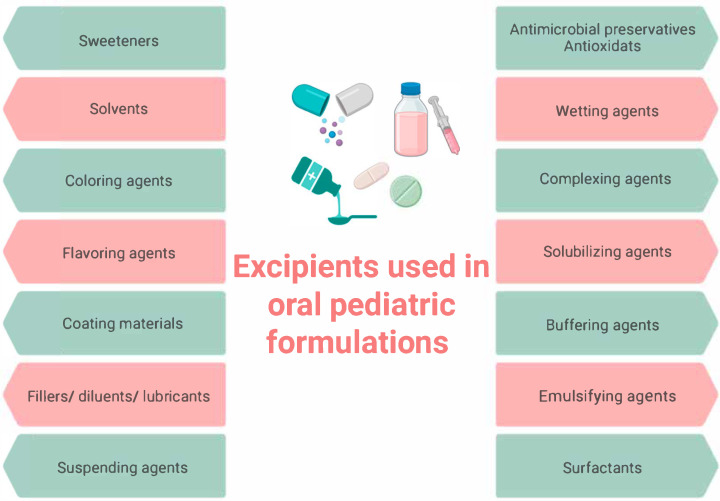
Examples of excipients used in oral pediatric formulations. Created with BioRender.com (accessed on 17 July 2025).

According to pediatricians from the American Pediatric Association, the unpleasant or bitter taste of drugs is the biggest barrier for completed treatment in pediatric practice [13]. Children are patients with specific taste preferences and taste correction plays a key role in the development of children’s dosage forms. For decades, oral liquid dosage forms such as solutions, syrups and suspensions have been affirmed as suitable for use in pediatric practice, especially by infants and preschool children. However, they also have disadvantages such as unpleasant taste, low stability (chemical, physical and microbiological) and are unsuitable for use in countries with a hot and humid climate [14,15]. Studies have shown that solid oral dosage forms, such as dispersible tablets, effervescent tablets, chewable tablets, and ODTs, are preferred for administration in young children [16]. These alternatives address several challenges associated with liquid formulations, including issues of safety, cost, and storage stability [17]. The selection of excipients in pediatric dosage forms, including preservatives, sweeteners, fillers, solvents, flavors, and colorants, presents a significant challenge (Figure 1) [18].

**Figure 2:**
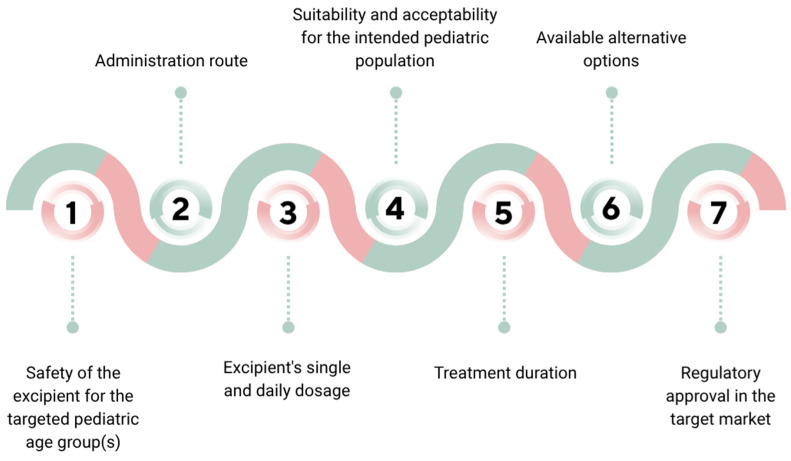
Factors influencing the choice of excipients in pediatric formulations. Created via Canva.com (accessed on 17 July 2025).

Excipients are chosen based on their functional roles and must be justified through a risk-based assessment that considers various factors, as highlighted in Figure 2 [19].

Evidence indicates that pediatric patients may develop adverse reactions not only to active pharmaceutical ingredients but also to certain excipients. To address this concern, the “KIDS List” (Key Potentially Inappropriate Drugs) was published in 2020, providing a list of both active substances and excipients considered potentially harmful in pediatric formulations [20]. These insights have informed the establishment of regulations governing the formulation and use of pediatric and neonatal medicines, mandating the disclosure of excipient presence and quantity in package leaflets, as well as specifying contraindications for excipients demonstrated to be toxic in children [21]. Based on these aspects, the STEP (Safety and Toxicity of Excipients for Pediatrics) database was created in 2012. This resource collects studies evaluating the toxicity of excipients not only in neonates and children, but also in adults [22,23].

Parental opinions can significantly influence children’s preferences regarding dosage forms. In addition to medical professionals, parents play a vital role in the acceptance of medications by pediatric patients. A triangular dynamic exists among children, healthcare providers, and parents, with the latter bearing the responsibility of facilitating children’s understanding and acceptance of treatment, while also managing the psychological stress associated with their child’s illness [24]. A study conducted in four European countries reported that over two-thirds of surveyed parents tend to give their children ODTs, particularly for allergic conditions requiring rapid onset of action [25]. ODTs represent a promising advancement in pediatric pharmacotherapy due to their improved taste, accurate dosing and easy administration. They are a novel alternative to traditional solid drug dosage forms and offer flexible administration for both pediatric and adult patients. These formulations disintegrate rapidly in the oral cavity upon contact with saliva, forming a suspension, solution, or soft paste that enhances mouthfeel and facilitates swallowing. This characteristic reduces the risk of choking often associated with conventional tablets or capsules [26]. ODTs are particularly acceptable in children over the age of five, especially when formulated with age-appropriate flavoring agents [27].

The development of an ‘ideal’ pediatric dosage form—characterized by a minimal drug dose, suitability for various age groups, reduced excipient content, pleasant taste, ease of absorption, and stability against external factors such as light, heat, and moisture—remains a significant challenge [4]. These requirements lead to a new trend among pediatric forms—the movement from liquid to solid dosed easily degradable forms [28].

Patient acceptability, safety, and accessibility must be carefully balanced, as the development of a single ‘ideal’ dosage form for all pediatric patients is rarely feasible. In many instances, selecting an age-appropriate formulation necessitates a compromise or the use of extemporaneous preparations to meet individual patient needs [29].

Key factors influencing the acceptability of pediatric medicines—which must be carefully considered during the design of new dosage forms—include palatability and ease of swallowing (e.g., size, shape, and texture), visual appeal (e.g., color, shape, embossing), taste, complexity of administration, required dose (e.g., volume or number of units), dosing frequency and treatment duration, mode of administration, administration device, container closure system, and any associated pain or discomfort [28].

This research aimed to investigate and analyze Bulgarian parents’ perspectives on their children’s preferences regarding different oral dosage forms, with a particular emphasis on ODTs. Additionally, challenges related to the development of age-appropriate formulations were comprehensively discussed.

## 2. Materials and Methods

### 2.1. Study Design and Participants

This study employed a cross-sectional design to assess parental perceptions regarding children’s preferences of different oral dosage forms. A randomly selected sample of Bulgarian parents residing in Plovdiv, the second-largest city in Bulgaria, was included. Eligible participants were required to be parents aged 18 years or older, have at least one child, and demonstrate adequate proficiency in reading and understanding the Bulgarian language.

### 2.2. Study Tool

The self-administered questionnaire used in this study was originally developed in Bulgarian by the research team, following an extensive review of the relevant scientific literature on pediatric dosage forms. An initial version of the questionnaire was reviewed by two independent qualified experts in pediatric drug formulations and pharmaceutical care to evaluate its accuracy, structure, content relevance, and language clarity. Based on their feedback, necessary modifications were made. To further ensure content validity, the revised version of the questionnaire was pilot tested with a small group of 10 parents of children. Minor modifications were made, including rephrasing and ordering items. Reliability was supported by ensuring consistency in item structure and wording and by pilot testing to confirm that parents interpreted the questions as intended. The final version comprised 18 items organized into 4 sections. Question formats included multiple-choice items (with options for more than one response) and binary (yes/no) items. The questionnaire required approximately 5 min to complete. The first section comprised questions focusing on children demographic characteristics (age, gender) and health status (the presence of chronic diseases, allergies, incidence of acute illnesses/conditions). The second section consisted of several questions regarding preferences and experience with oral dosage forms (preferred formulation; difficulties in administrating; favorite flavor, etc.). The third section encompassed questions concerning participants’ perceptions of ODT usage (general knowledge and previous usage). The last section included questions related to experiences of adverse drug reactions (ADRs) due to pharmaceutical excipients, use of pediatric oral extemporaneous preparations and the need for additional information. A consent statement was included at the beginning of the questionnaire, detailing the purpose of the study, estimated completion time, confidentiality of responses, and the voluntary nature of participation.

### 2.3. Data Collection

Data collection took place between January and March 2021. The questionnaire was administered online via the Google Forms platform, and the link was distributed through parenting support groups on social media platforms like Facebook, WhatsApp, Viber and Instagram. Participation was accessible to any individual who met the eligibility criteria and engaged with the survey link. Since the study was conducted during the COVID-19 pandemic, this recruitment process was chosen to reach the large and different population of parents. The sample size was not predetermined through statistical power calculation, as the study had an exploratory character. Instead, participation remained open throughout the data collection period, resulting in a final sample deemed sufficient to observe meaningful trends and descriptive patterns. Participants were provided with an informed consent statement and detailed information about the study on the first page of the questionnaire. The parents were apprised that their consent to participate was signified by an affirmative response to the initial question.

### 2.4. Ethical Approval

The study protocol was reviewed and approved by the Ethics Committee of the Medical University of Plovdiv (Protocol No. 1/22 February 2018).

### 2.5. Statistical Analysis

Statistical analysis was conducted using IBM SPSS Statistics version 19 and Microsoft Excel for Windows 10. Descriptive statistics were applied, and results were presented as frequencies and percentages. Statistical comparisons were performed using chi-square tests, with a *p*-value of <0.05 considered statistically significant.

## 3. Results

Table 1 summarizes demographic and health-related characteristics of children according to their parents’ answers.

The mean age of the children was 6.5 years (SD ± 4.4), ranging from 1 to 18 years, and more than half of them were boys (59.4%, *n* = 180). According to their age, children were classified into three groups. The majority of children belonged to the age group 0–6 years (58.4%), followed by 7–12 years (26.7%), and the remaining 14.9% were in the group 13–18 years. Only 4.0% of children had chronic disease and 13.9% of parents reported allergies. The most frequently reported allergens included house dust, pollen, dust mites, animal dander, medications, and foods (e.g., eggs, milk, fish, gluten, nuts, and fruit), as well as cigarette smoke and perfumes. Several parents reported multiple concurrent allergies. The frequency of acute illnesses or conditions among children, as reported by parents, varied. The majority of respondents (72.3%) indicated that such illnesses occurred several times annually, followed by 17.8% who indicated occurrences every two months, and only 2.0% reported occurrences on a weekly basis.

**Figure 3:**
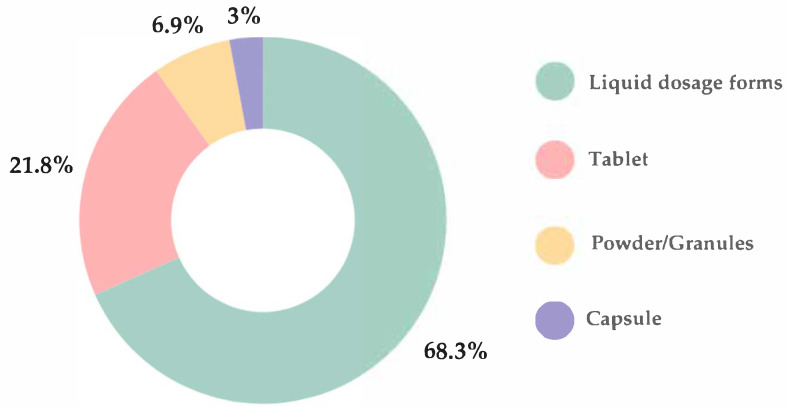
The most preferred oral dosage forms according to parental answers.

The most preferred oral dosage forms were liquid (68.3%), including syrups, solutions, suspensions, and emulsions, followed by tablets (21.8%), powders/granules (6.9%), and capsules (3%) (Figure 3). A statistically significant difference in dosage form preferences was observed between healthy children and those with allergies (*p* < 0.001), with the latter group demonstrating a greater preference for tablets over liquid formulations.

There were no statistically significant association between child’s gender and preferred oral dosage form (*p* = 0.189). Liquid formulations were the preferred oral dosage form among the youngest children, with 72.5% of those aged 1–6 years favoring this option. However, preference for liquids declined progressively with age, reported by only 23.2% of children aged 7–12 years and 4.3% of those aged 13–18 years.

**Figure 4:**
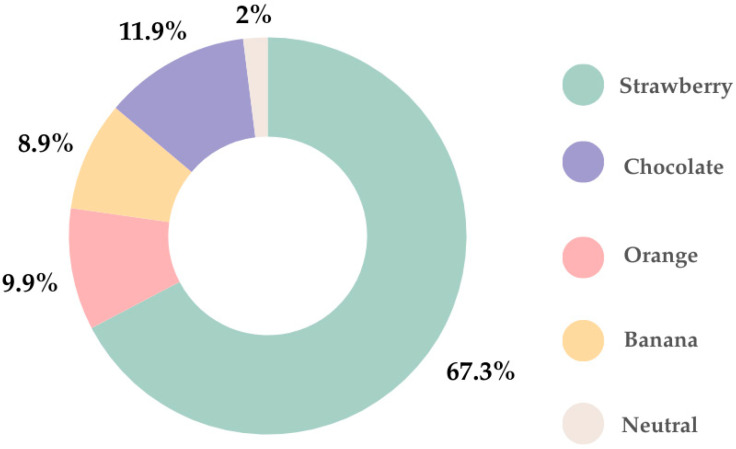
Children’s preferred flavors.

In regard to flavor, the favorite was strawberry (67.3%), followed by chocolate (11.9%). Only 2% of parents indicated that their children preferred neutral flavor (Figure 4).

Table 2 presents parental responses regarding factors influencing oral dosage forms intake in children.

Just over one-fifth of parents (21.8%) reported encountering difficulties when administering medications to their child. There was no statistically significant association between the frequency of the child’s acute illnesses and whether they experience difficulty taking medication (*p* = 0.110). Parents of children with allergies reported significantly greater difficulties in administering medications compared to parents of healthy children (*p* < 0.01). The majority of parents (85.1%) indicated that the taste of the oral dosage form was an important factor for their child. Just over half of parents (51.5%) reported having to divide conventional tablets to facilitate the administration process for their child.

Participants’ perceptions of ODT usage, including general knowledge and previous experience, are summarized in Table 3.

The majority of respondents (82.2%) reported having heard of ODTs. Despite this high percentage, only 11.2% indicated that their child had previously taken ODTs. More than half of parents (66.3%) stated their child had not taken ODTs, and 22.4% were uncertain. Parental opinion regarding ODTs was very positive, with 91.1% expressing a preference for their child to take ODTs instead of conventional tablets.

**Figure 5:**
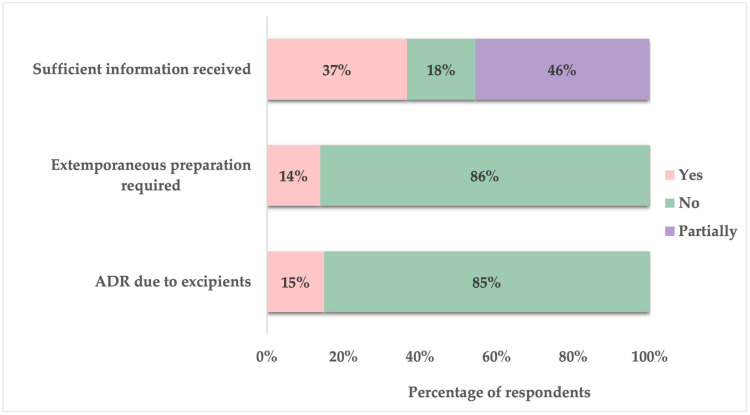
Parental responses regarding ADRs, extemporaneous preparations, and satisfaction with medication-related information received from healthcare providers for pediatric oral dosage forms.

Approximately 15% of parents reported that their child had experienced an ADR due to pharmaceutical excipient included in the oral dosage form. Additionally, 14% indicated that it had been necessary for their child’s medication to be prepared as an extemporaneous formulation in a pharmacy. When asked about the medication-related information received from healthcare providers (physicians and pharmacists) regarding their child’s oral medications, only 37% of parents felt sufficiently informed, while 46% stated that they only partially received adequate information. The remaining 18% reported not receiving enough information (Figure 5).

## 4. Discussion

### 4.1. Challenges in Developing Age-Appropriate Pediatric Formulations

According to EMA guidelines from 2023 the development of pediatric formulations is increasingly focused on age-appropriate dosage forms that prioritize acceptability, safety, and the ability to deliver precise and adjustable doses based on a child’s needs [10]. Additionally, these formulations must ensure acceptable palatability, include suitable excipients, and comply with regulatory standards [9,30]. While taste and palatability are the most researched aspects of pediatric medicine acceptability, there are still no standardized methods or criteria to define what constitutes an acceptable taste for pediatric oral formulations or which flavors children prefer. Establishing a clear methodology or standard is essential to support palatability claims for pediatric products [31].

The International Conference on Harmonization of Technical Requirements for the Registration of Pharmaceuticals for Human Use endorsed by the World Health Organization (WHO) divided the pediatric group as follows: preterm neonates, full-term newborn infants (birth until 27 days), infants and toddlers (28 days until 23 months of age), children (2 years until 11 years of age), and adolescents (12 years until 16–18 years of age) (depends on region) [1]. Pediatric patients cannot simply be regarded as smaller versions of adults; it is essential to determine the appropriate dosage, safety, and efficacy of a medicine specifically for their age group [32].

In its key pharmaceutical development guideline, the EMA defines an age-appropriate pediatric medicine as one whose pharmaceutical design is suitable for the intended age group(s). This design includes factors such as formulation composition, dosage form, dosing frequency, and packaging. According to the EMA guideline, tablets for children under 6 should not exceed 5 mm in size. However, even this can be challenging for many, making liquid or orally disintegrating forms a better option. By age 6 and beyond, acceptance of small to medium-sized tablets improves, though a notable portion of children still struggle with swallowing solid dosage forms. For treatments covering a broad age or weight range, offering flexible dosage strengths may be necessary [11].

It has been noted that children of varying ages and abilities require tailored dosage forms to suit their capabilities, such as their ability to safely and confidently swallow tablets. Studies have consistently shown that smaller tablets are easier for young children to manage. Additionally, the use of small particulates combines several benefits of both liquid and solid medicines, making it a promising technology for developing pediatric dosage forms [3].

According to the results of our survey, the two most preferred oral dosage forms were liquids (68.3%) and tablets (21.8%). These findings align with a questionnaire-based study conducted across six European countries by the European Paediatric Translational Research Infrastructure (EPTRI), which also identified liquids and tablets as the most commonly preferred formulations [33]. Furthermore, this study indicated that liquid formulations were predominantly selected by children under 12 years of age and by those infrequently taking medications or considered healthy [33]. Similarly, in our research, liquid formulations were the most preferred among children aged 1 to 6 years and those without allergies. A nationwide survey in China assessing the perspectives of children and their caregivers regarding pediatric medicines and devices revealed that the majority of participants (83%) used oral dosage forms, with granules, syrups, and tablets ranking as the top three most frequently used formulations [34]. Additionally, an anonymous observational questionnaire conducted in Japan among children and their parents or caregivers found that powders were most commonly prescribed for children under 10 years of age, followed by liquids [35]. According to Alessandrini et al., taste and swallowability were identified as the two most influential attributes affecting the selection of most favorite medications [33]. Similarly, 85.1% of parents participated in our survey indicated that the taste of the oral dosage form was an important factor for their child.

In December 2007, the WHO introduced the “Make Medicines Child Size” initiative to increase awareness and drive action toward better availability and access to child-specific medicines. The WHO Model Formulary for Children 2010 offers independent prescriber guidance on dosage and treatment, based on the WHO Model List of Essential Medicines for Children, which was first created in 2007 and is reviewed and updated biennially [19].

For children under 2 years old, liquid dosage forms are generally preferred, though orally disintegrating formulations may also be suitable in some cases. Liquid dosage forms are generally preferred for young children, despite certain challenges. Suspensions must be shaken before administration to ensure accurate dosing and multidose packaging should include a measuring device to dispense the appropriate amount based on the child’s age and weight. To maintain microbiological quality, liquid formulations require preservatives, which must be carefully selected for safety [36]. Taste is a crucial factor in pediatric acceptance, often necessitating taste-masking techniques. Many solid oral dosage forms, such as film-coated tablets (e.g., amoxicillin) and capsules (e.g., clindamycin), present taste challenges due to the bitterness of the active ingredient.

Due to the lack of age-appropriate dosage forms, tablets are the most commonly used in pediatric practice, often requiring modifications such as splitting, dispersing, or crushing to facilitate administration [37]. The EMA generally advises against splitting or crushing solid dosage forms to achieve the correct dose, as the active pharmaceutical ingredient (API) may not be evenly distributed unless the validation process ensures uniformity. Between ages 2 and 6, a child’s ability to swallow small tablets or capsules varies greatly, often depending on prior experience with medications. Half of the parents who took part in our survey reported the need to split conventional tablets to facilitate administration to their child.

Commercially available age-appropriate formulations may not always provide the diverse range of doses needed for neonatal and pediatric patients. To address this challenge, medications are often modified by physically altering the dosage form to achieve the necessary dose for administration. Manipulation refers to the physical modification of a drug dosage form to obtain and deliver the required portion of the drug dose [38].

### 4.2. Extemporaneous Preparations

When essential drugs for preparing individualized pediatric dosage forms are unavailable, the only option is extemporaneous compounding using commercially available pharmaceutical products [39]. Extemporaneous dispersing or compounding plays a crucial role in pediatric practice, enabling the preparation of age-appropriate dosage forms when suitable authorized medications are unavailable. The process of compounding carries inherent risks, and alternative approaches such as dose rounding, therapeutic substitution, or modifying adult dosage forms may be considered [40].

Extemporaneous dispensing can be minimized if healthcare professionals collaborate to ensure the availability of commercially produced age-appropriate dosage forms whenever possible. This includes increasing prescribers’ awareness of locally available formulations, promoting rational prescribing, and utilizing therapeutic substitution and dose rounding when necessary. The pharmaceutical industry can support these efforts by offering affordable pediatric dosage forms and validating the safe manipulation or compounding of their adult formulations when appropriate [40].

Most of the extemporaneous medicines administered to pediatric patients are prepared by pharmacists. There is a restricted ability to ensure quality control in pharmacies, as they often lack the necessary analytical equipment [37,41]. Their bioavailability, suitability, stability, and safety could not be proved. The preparation of suitable pediatric dosage forms extemporaneously can be achieved either by utilizing an API, if available, or by modifying commercially available drug products. Some marketed drug formulations require manipulation to achieve the appropriate pediatric dose, such as crushing tablets or mixing them with food in ways not specified in the Summary of Product Characteristics. However, this practice often disregards potential changes in pH, viscosity, fat, and sugar content, which may impact drug bioavailability [42]. This off-label use of medications lacks the rigorous regulation and quality control of licensed medicinal products, potentially compromising treatment efficacy and patient outcomes. A major problem is the absence of preclinical tests and clinical trials for many pediatric medicines, making it difficult to ensure their safety. This increases the risk of ADRs, drug interactions, and ineffective treatment due to inaccurate dosing [43].

The International Pharmaceutical Federation (FIP) conducted a global survey to assess pediatric oral extemporaneous compounding practices. A total of 479 participants actively engaged in compounding across all WHO regions completed the survey. The findings revealed that over 90% of respondents reported preparing oral liquid formulations. Regarding the use of flavoring excipients, 57% of pharmacists in Europe indicated that they do not incorporate flavoring agents in their extemporaneous preparations [44].

The preparation of extemporaneous oral medications for pediatric patients is a common practice in both hospital and community pharmacy settings throughout Europe. Additionally, 14% of the participants in our study reported that their child’s medication was prepared as an extemporaneous formulation in a pharmacy.

### 4.3. Excipients in Pediatric Medicines

Designing a suitable dosage form is a major challenge in pediatric drug delivery research, as it impacts key factors such as excipient selection and the need for additional administration devices [45]. Almost all drug formulations contain excipients that are tested for quality according to official standards. However, these standards are mainly made for adults and do not always guarantee safety or suitability for children [42].

Excipients are used to develop suitable dosage forms that make medicines easier to administer, improve usability, and promote treatment adherence. They can serve simple purposes, like acting as fillers, or perform more advanced functions, such as regulating how a drug is released in the body [3]. It is essential to simplify pediatric formulations by minimizing both the number and quantity of excipients while ensuring the product retains its required functionality. The selection of excipients must be carefully justified, prioritizing safety for the intended pediatric population [6].

While excipients are often considered inert, many can exert effects on the body, and their interaction mechanisms may differ significantly between adults and children of different age [3]. Despite notable differences in pharmacokinetics and pharmacodynamics between adults and pediatric patients, it is often assumed that excipients deemed safe for adults are also suitable for neonates and children. However, in recent years, regulatory authorities have highlighted growing concerns that certain excipients may be less well tolerated in children, particularly neonates, whose physiological systems are still maturing [42]. Thus, both the choice of an appropriate formulation and the selection of excipients are crucial in developing suitable pediatric dosage forms. The EMA guideline provides guidance on how to ensure the safe selection of excipients [11].

Since 2001, the European Study of Neonatal Exposure to Excipients (ESNEE) has been investigating the toxicity of excipients in newborns [46,47]. As part of this work, a priority list of excipients identified as potentially toxic has been created, and additional studies are ongoing to assess neonatal exposure levels.

Recently, the European Pediatric Formulation Initiative, in collaboration with NICHD and the US Pediatric Formulation Initiative, introduced the Safety and Toxicity of Excipients in Pediatrics (STEP) database [23]. This resource aims to provide valuable information on the safety and toxicity of excipients used in pediatric medications [48].

When developing databases on the safety and toxicity of excipients in the pediatric population, priority is given to those most likely to cause harm or adverse effects in this group [49]. Special attention must be given to selecting excipients such as sweeteners, flavors, plasticizers, solvents, preservatives, and colorants in medicinal formulations. Examples include Propylene glycol (PG), Ethanol, Polysorbate 80, Benzyl alcohol, Parabens (propyl, methyl, ethyl and butyl), Benzalkonium chloride, Aspartame, Sorbitol, Benzoic acid and Sodium benzoate.

Sweeteners in pediatric formulas are restricted due to potential side effects. In the United States, certain sweeteners like cyclamates are banned, while others, such as saccharin, require warning labels, necessitating careful formulation for compliance. Aspartame, an artificial sweetener, poses risks for individuals with phenylketonuria and certain genetic conditions and may trigger cross-reactions with sulfonamides [32].

Excessive sucrose should be avoided as it lowers dental plaque pH, leading to enamel erosion and dental caries. Similarly, high fructose intake can have a laxative effect on children. Sorbitol and xylitol may cause osmotic diarrhea, though xylitol also helps prevent cavities. Lactose must also be used cautiously, as lactose intolerance is becoming more common in children [32].

Ethanol and PG are commonly used as solvents in oral liquid formulations. However, their use in pediatric products raises toxicity concerns. Ethanol-containing formulations may interact with certain drugs, affecting their absorption or metabolism and potentially leading to drug interactions [42].

PG can accumulate in the body due to the underdeveloped metabolic pathways in children under four years old, particularly the limited activity of alcohol dehydrogenase. Its primary toxic effect is depression of the central nervous system. Therefore, products with high levels of PG are not suitable for young children, especially those under four years of age [32].

Special attention is required when incorporating preservatives into medicines for newborns and young infants, as their use in infant dosage forms is already restricted by law. For instance, neonates and infants have immature metabolic systems, which can result in the accumulation and toxicity of excipients like propylene glycol and benzoates; therefore, their use should be approached with caution in very young patients [30]. Whenever possible, preservative-free formulations should be prioritized. Rather than developing multidose liquid formulations that require preservatives, alternatives such as solid dosage forms—mini-tablets, orodispersible tablets, granules, granulates, or powders in sachets that can be reconstituted into liquid immediately before use—should be explored. If preservatives are necessary, their concentration should be kept to the lowest effective level [3]. Flavoring agents are often added to enhance the taste of oral formulations and improve patient adherence, but they also pose potential risks of allergy and sensitization [50]. When children are given extemporaneous (specially prepared) medicines, they may be exposed to high levels of certain excipients, which can also cause toxicity [28].

From a safety perspective, solid oral formulations may be preferable to liquid forms, as they eliminate the need for antimicrobial preservatives. Additionally, solid dosage forms may reduce the risk of unpleasant taste since the drug remains less dissolved at the time of administration, making sweeteners and flavoring agents unnecessary. On the other hand, masking the taste in liquid formulations, as well as in dispersible solids like chewable or orodispersible tablets, can pose considerable technical challenges [51].

### 4.4. Taste Masking

The oral route is the preferred method of drug administration for pediatric patients and their caregivers due to its convenience. However, the bitterness of many medications poses a significant challenge, leading to noncompliance issues. Parents and hospital caregivers often struggle with administering bitter drugs, which can result in refusal, missed doses, or the need for repeated dosing due to spillage or vomiting. This nonacceptance can have a serious impact on the effectiveness of the overall treatment regimen [32].

Pediatric medications must be palatable to ensure proper dose acceptance and patient adherence. A palatable drug product is one in which unpleasant sensory attributes have been minimized or eliminated—meaning it is not excessively bitter, has a smooth mouthfeel, and lacks any noticeable unpleasant odors [36]. It is important to acknowledge that palatability extends beyond taste, as factors like smell and texture also play a significant role and should not be overlooked [31].

To make the medicines more palatable and easier to administer, patients and caregivers often mix them with food or beverages to dilute or mask their taste. However, this method poses certain risks, such as incomplete consumption due to an excessive volume or inadequate taste masking [32]. As a result, relying on food or beverages as the primary means of taste masking is not ideal [50]. Instead, effective taste-masking techniques should be incorporated during the formulation and preparation process rather than applied afterward [52]. Taste-masking methods include adding sweeteners and flavoring agents, increasing viscosity, or incorporating cosolvents to reduce the perception of bitterness. However, these conventional techniques may not be effective for all bitter drugs [32].

Children’s taste preferences differ significantly from those of adults, with a strong preference for sweet, candy-like flavors. Since the natural taste of APIs and excipients can be unpleasant, the EMA has issued a list of recommended flavors based on the original taste of the API (such as acidic, alkaline, bitter, salty, or sweet) [48]. Additionally, certain flavors are commonly linked to specific treatments, such as lemon or orange for vitamins and mint for stomach pain relief [24].

The findings of our study underscore the importance of palatability, with taste emerging as a primary determinant of acceptability among children. According to parental responses, liquid dosage forms remain the most preferred option for younger children, with sweet, fruit-flavored formulations being particularly favored. A study conducted by Walsh et al. shows that the approval of drug therapy by children varies from 11% to 93% and the most important influencing factors are the type of dosage form and its taste [50]. The children have a preference not only to liquid dosage forms such as solutions, suspensions and syrups, but also for ODTs or chewable tablets. They accept sweet and salty taste and completely reject the bitter one, because they have a low tolerance to unpleasant or tasteless drugs. On the other hand, the pleasant taste can be a cause of intoxication due to a higher dose. Therefore, the “neutral” taste is accepted as the most preferred taste sensation [12]. The taste of the drug can be felt only if it dissolves in the oral cavity due to the saliva or other liquid or is administrated as a solution that comes into contact with taste buds. In oral dosage forms, in which the drug remains undissolved in the oral cavity, taste sensations cannot be induced because it does not come into contact with taste receptors [50].

More advanced approaches involve complexation with molecules like cyclodextrins, which encapsulate the bitter compound, or ion exchange resins that modify drug interactions by exchanging ions. Additionally, barrier coating with polymers, lipids, or other inert materials provides an effective way to conceal bitterness and improve palatability [32].

### 4.5. Orodispersible Tablets (ODTs)

Addressing the challenges of swallowing solid dosage forms involves exploring orodispersible formulations, such as ODTs. These uncoated tablets are designed to rapidly disperse in the oral cavity before being swallowed. ODTs can be formulated as immediate-release or modified-release systems incorporating microparticles, pellets, or granules coated with functional excipients. Currently, they are primarily manufactured through direct powder compression, though innovative techniques like 3D printing are also being utilized in their production [53].

The Children’s Acceptability of Oral Formulations (CALF) study of Ranmal et al. explored end-user perceptions and practices regarding solid oral dosage forms, including tablets, capsules, chewable, ODTs, multiparticulates (taken with food), and mini tablets (administered directly into the mouth). Attitudes toward these dosage forms varied mainly by age and prior experience. Preference for tablets and capsules increased with age, peaking around 14 years. Swallowing whole tablets can be challenging, particularly for pediatric patients. Chewable and orodispersible formulations were generally favored across all age groups, whereas multiparticulates were perceived as less desirable [45]. Orodispersible formulations are better for patients with difficulty swallowing, such as young children, as they are stable and quickly dissolve in the mouth with saliva, eliminating the need for chewing or water intake [54]. ODTs were the first solid formulation successfully introduced for children. While they help address swallowing difficulties, they still lack dose flexibility, similar to conventional tablets. Additionally, their low hardness necessitates specialized packaging to prevent breakage during handling and transport. They are deemed suitable for low- and middle-income countries due to their advantages over liquid formulations, including greater stability and reduced bulk. Once dispersed, they are well-tolerated by patients of all ages, including those with swallowing difficulties, while still retaining the benefits of solid oral dosage forms [30].

There has been a significant shift toward solid formulations over liquids due to concerns with stability, palatability, and cost. Advances in innovative technologies, including orodispersible, multiparticulate, and chewable formulations, combine the benefits of solid dosage forms with the flexible dosing and ease of ingestion typically associated with liquids.

For children under 2 years old, orodispersible formulations provide greater ease of ingestion, while orodispersible mini-tablets or multiparticulates can enhance dose flexibility and accommodate a wider dosing range when needed. Multiparticulates and mini-tablets should be assessed for administration either with food or through appropriate age-specific devices or packaging [45]. ODTs could be prepared using various techniques, including direct compression, lyophilization, flash heat processing, tablet molding, and, more recently, 3D printing technology. However, direct compression and lyophilization remain the most commonly used manufacturing methods.

ODTs are often highlighted as highly promising, child-friendly dosage forms. In a study by Alyami et al. involving 104 children aged 6–18 years, ODTs emerged as the most preferred option, with 58% selecting them as their favorite [55]. ODTs are primarily used for school-aged children (over 6 years old), but some evidence shows that they are also generally well accepted by preschool-aged children. The study by Wiedeley et al. conducted in 2021 found no evidence supporting restrictions on the use of ODTs in children [56]. It is important to recognize that healthcare professionals’ assumptions about a minimum age for ODT use are not based on actual data [56]. The global ODT market is projected to reach USD 9.29 billion by 2025, driven by factors such as the rising prevalence of chronic diseases, ease of use, improved patient adherence, and advances in drug delivery technologies [57]. The global orally disintegrating tablet (ODT) market is projected to expand at a compound annual growth rate (CAGR) of 8.15% between 2025 and 2034. In 2024, North America accounted for the largest regional market share, representing 37% of the total [57]. Current trends suggest that ODTs will increasingly support personalized medicine. Their growing popularity is also linked to effective taste-masking strategies, the introduction of new formulations, and the advantage of rapid drug action following dissolution.

Our findings show that although 249 parents stated being familiar with ODTs, only 11.2% reported that their child had previously taken this dosage form. This gap suggests a disconnect between awareness and actual experience. A study conducted by Alessandrini et al. reported that ODTs were among the least selected options by both parents and children in terms of most and least favorite dosage forms, which indicated a lack of knowledge regarding this dosage form [33]. In another study by Orlu et al., which assessed the acceptability of orodispersible films in pediatric patients, 83% of caregivers were unfamiliar with ODFs prior to participation [58]. However, existing acceptability studies have reported positive responses and favorable preferences toward orodispersible formulations from both children and their caregivers [58,59]. Consistent with these findings, 91.1% of parents in our study expressed a preference for their child to receive ODTs over conventional tablets.

Flexible dosage forms, such as sachets and ODTs, which can also be used to prepare oral liquids, are generally the most suitable and should be prioritized, especially for younger children. These formulations are considered appropriate for various APIs, provided they do not require precise dose titration or belong to poorly soluble BCS classes. If a medicine can be dispersed in breast milk, it may also be suitable for very young infants under six months of age [19]. The innovative 3D-printed medicinal gummies, customized for individual patients, could serve as a suitable oral dosage form for pediatric patients due to their suitable appearance and pleasant taste and texture [60].

### 4.6. Strengths and Limitations

To our knowledge, this was the first study in Bulgaria to evaluate parents’ perspectives on their children’s preferences regarding different oral dosage forms.

Our study has several limitations that should be mentioned. Firstly, the current study employed a cross-sectional design which does not let any causal relationships be assessed. Moreover, cross-sectional studies typically utilize questionnaires for data collection, which may lead to inaccuracies due to the inability to independently verify participants’ responses (reporting bias). In addition, self-reported information is susceptible to recall bias and social desirability bias, both of which can compromise the reliability and validity of the findings. The use of self-reported data introduces susceptibility to recall (memory) bias, as parents may not accurately remember past experiences. Secondly, no data were collected regarding the specific chronic illnesses affecting the children or whether they are on daily medication. Additionally, the study did not include information about the APIs present in ODTs. Socio-demographic data related to parents—such as age, employment status, and household income—were also not collected. Furthermore, the study was conducted in a single city in Bulgaria, which restricts the extent to which the findings can be generalized to parents from other regions. We acknowledge the potential for selection bias due to the recruitment method. Since the survey was distributed via social media, parents who are active internet and social media users were more likely to participate. Consequently, the sample may not fully represent parents with limited internet access, lower digital literacy, or those not engaged in online parenting groups.

Despite these limitations, the research offers valuable perspectives on parental opinions concerning pediatric oral dosage formulations and has the capacity to form an essential framework for future investigations with more comprehensive and diverse groups.

## 5. Conclusions

Developing the “ideal” pediatric dosage form is a complex and multifactorial process, associated with a numerous challenge and dependent on various factors. The results of our study show that the taste of the dosage form is a leading factor in child acceptability. Liquid dosage forms remain the preferred alternative among younger children according to their parents, with sweet, fruit-flavored formulations being particularly favored. Parental opinion regarding ODTs was very positive, with 91.1% expressing a preference for their child to take ODTs instead of conventional tablets. Given the common difficulties associated with swallowing solid oral dosage forms, ODTs represent a promising alternative that is generally well-accepted across various pediatric age groups. Future research involving direct input from children may offer a more comprehensive understanding of their preferences regarding oral dosage forms.

## Figures and Tables

**Table 1:** Demographic and health-related characteristics of children.

Characteristics	Number, *n*	Percentage, %
**Child’s age**		
0–6	177	58.4%
7–12	81	26.7%
13–18	45	14.9%
**Gender**		
Boy	180	59.4%
Girl	123	40.6%
**Chronic disease**		
Yes	12	4.0%
No	291	96.0%
**Allergies**		
Yes	42	13.9%
No	261	86.1%
**Frequency of acute illnesses**		
Weekly	6	2.0%
Monthly	24	7.9%
Every two months	54	17.8%
Several times per year	219	72.3%

**Table 2:** Parental responses on factors affecting oral dosage forms intake in children.

Item	Yes, *n* (%)	No, *n* (%)
Do you encounter difficulties when administering medications to your child?	66 (21.8%)	237 (78.2%)
Is the taste of the oral dosage form an important factor for your child when taking medication?	258 (85.1%)	45 (14.9%)
Do you need to divide conventional tablets to make them easier for your child to take?	156 (51.5%)	147 (48.5%)

**Table 3:** Parents’ perceptions of ODT usage.

Item	Number, *n*	Percentage, %
**Have you heard of ODTs that disintegrate in the mouth upon contact with saliva, without the need for water?**		
Yes	249	82.2%
No	54	17.8%
**Has your child ever taken ODTs?**		
Yes	34	11.2%
No	201	66.3%
Not sure	68	22.4%
**Would you prefer your child to take ODTs instead of conventional tablets?**		
Yes	276	91.1%
No	27	8.9%
**In your opinion, at what age are ODTs appropriate for children?**		
Over 2 years	63	20.8%
Over 3 years	99	32.7%
Over 4 years	30	9.9%
Over 5 years	111	36.6%

## Data Availability

Data are contained within the article.

## Abbreviations

The following abbreviations are used in this manuscript:

ADR	Adverse drug reaction
API	Active pharmaceutical ingredient
EMA	European Medicines Agency
ODT	Orodispersible tablet
PG	Propylene glycol
WHO	World Health Organization
